# Self-Construal as a Predictor of Antagonistic Personality Traits

**DOI:** 10.3390/bs16010091

**Published:** 2026-01-08

**Authors:** Bonnie Simpson, Julie Aitken Schermer

**Affiliations:** 1 DAN Department of Management and Organizational Studies, Faculty of Social Science, The University of Western Ontario, 1151 Richmond Street, London, ON N6A 5C2, Canada; 2 Department of Psychology, Faculty of Social Science, The University of Western Ontario, 1151 Richmond Street, London, ON N6A 5C2, Canada

**Keywords:** sadism, antagonistic tetrad, psychopathy, narcissism, Machiavellianism, self-construal, interdependence, independence

## Abstract

Self-construal refers to how individuals perceive themselves relative to others and includes dimensions of independence and interdependence. To understand antagonistic personality traits of Machiavellianism, psychopathy, narcissism, and sadism, we investigate how these four traits (the Antagonistic or Dark Tetrad) are predicted by independent and interdependent self-construal using self-report measures from 861 Canadian university students. Direct entry linear regression found that after accounting for age and gender, each antagonistic trait was significantly predicted by independent and interdependent self-construal. As the pattern of regression weights differ for self-construal dimensions, we conclude that self-construal differentially predicts the Antagonistic Tetrad. These results add to work examining how self-construal is related to the Antagonistic Triad by the addition of sadism and investigating the predictive ability of self-construal.

## 1. Introduction

Antagonistic personality traits reflect a tendency to disregard others’ needs and pursue self-interest through manipulative, callous, or attention-seeking behaviours (Wright, 2019). In the present research, we focus on traits traditionally labelled as the Dark Tetrad—Machiavellianism, narcissism, psychopathy, and sadism—which can also be conceptualized more neutrally as antagonistic personality traits (i.e., the Antagonistic Tetrad; Kay et al., 2025). Understanding how these traits relate to self-construal—the way individuals define themselves in relation to others—is theoretically important because self-construal shapes the cognitive, motivational, and interpersonal processes through which personality is expressed (Cross et al., 2011; Morf & Rhodewalt, 2001). Independent self-construal, characterized by self-focus, personal goal pursuit, and competitive orientation (Markus & Kitayama, 1991; Oyserman et al., 2002), may align with the self-serving, instrumental strategies found in Machiavellianism or certain expressions of narcissism. Interdependent self-construal, emphasizing group harmony and relational obligations, may dampen overt antagonism but can, in some contexts, enable socially embedded forms of manipulation (Lalwani & Shavitt, 2013). These nuanced pathways suggest that self-construal may differentially predict the expression of antagonistic personality traits, making it a meaningful construct for advancing theory on the social–cognitive foundations of personality. The present study tests this proposition by examining whether independent and interdependent self-construals predict variation in the Antagonistic Tetrad.

Understanding antagonistic personality traits has valuable theoretical and practical implications across fields of research, including clinical (Jain et al., 2023), social (e.g., Forsyth et al., 2021), and organizational psychology (e.g., Thibault & Kelloway, 2020), and consumption (e.g., Bonfá-Araujo et al., 2023; Konuk & Otterbring, 2024). The current study expands on the prior research in two ways. First, we assess the Antagonistic Tetrad, incorporating the addition of sadism which has not been considered in prior work that looks at antagonistic personality traits and self-construal. Second, building on work which examines correlations between self-construal and the dark triad, we test the predictive ability of both independent and interdependent self-construal on each of Machiavellianism, narcissism, psychopathy, and sadism. Understanding how different self-construals predicts these traits bridges personality theory with cultural frameworks and can reveal insights into how cultural orientations shape personality expressions and explore potential for both cultural moderators of antisocial behaviours and development of intervention strategies.

### 1.1. The Antagonistic Tetrad

Personality traits previously labelled as the Dark Tetrad—Machiavellianism, narcissism, psychopathy, and sadism—have more recently been conceptualized more neutrally as antagonistic personality traits (Chester et al., 2025) and referred to as the Antagonistic Tetrad. Antagonism reflects a tendency toward low agreeableness, including callousness, manipulativeness, and competitive or oppositional interpersonal behaviour. All four Antagonistic Tetrad traits share this antagonistic core, although sadism additionally involves the hedonic enjoyment of others’ suffering, representing a specific manifestation of antagonism. Framing the Dark Tetrad as antagonistic traits allows for a theoretically grounded, descriptive perspective that emphasizes interpersonal dispositions and personality mechanisms, rather than moral evaluation, while preserving the ability to investigate individual differences in socially aversive behaviours.

In this research, we consider the Antagonistic Tetrad, four antagonistic personality dimensions that are related but differ in how they correlate with other variables. We include subclinical psychopathy (reflecting callousness, erratic behaviour, and antisocial tendencies), Machiavellianism (a desire to manipulate others and a generally cynical attitude), and narcissism (an extremely positive or possibly inflated sense of the self). Past research has suggested that although these constructs may share a common core, possibly reflecting callousness and manipulation (Furnham et al., 2014), narcissism has been suggested to be more distinct than Machiavellianism and psychopathy, as it captures the social–interpersonal aspect of malevolent personality (Egan et al., 2014; Kowalski et al., 2016; Muris et al., 2017). Building on this research, Buckels et al. (2013) proposed incorporating everyday sadism into a ‘Dark Tetrad’ of personality, reflecting the enjoyment of hurting others and observing acts of aggression (Paulhus et al., 2021). While this extension has been influential, it has also received theoretical critique, particularly regarding the distinctiveness of sadism, with some research suggesting substantial overlap with psychopathy (Blötner & Mokros, 2023) and others highlighting the empirical distinction of sadism (Kay et al., 2025). These four Antagonistic Tetrad dimensions have been reported to intercorrelate significantly (Brook et al., 2016) yet exhibit distinct properties (Međedović & Petrović, 2015). In the present study, our primary aim is not to validate or challenge the theoretical structure of the Antagonistic Tetrad, but rather to examine how independent and interdependent self-construal dimensions predict variation across these antagonistic personality traits.

### 1.2. Self-Construal

One aspect of interest regarding the Antagonistic Tetrad is how these personality dimensions might be formed by representations of the self. In this research, we examine self-construal (Markus & Kitayama, 1991) as one means of conceptualizing how individuals define and understand the self. Independent and interdependent self-construal are two distinct dimensions of self-construal. In other words, one way of thinking about the self is to consider that some individuals prioritize an independent self: seeking independence, autonomy, and separation from others; whereas others prioritize a more interdependent self: seeking to maintain harmony in their groups and fit in with others (Cross et al., 2011). Individuals possess both dimensions, and cultural context often promotes one dimension more than the other, leading individuals to have a more dominant self-view (Markus & Kitayama, 1991; Singelis, 1994; Triandis, 1989). Importantly, self-construal is not conceptualized as a unipolar continuum (e.g., self-focus to other-focus) but rather as two separate dimensions, meaning that individuals might be high on one, the other, both, or neither.

#### Self-Construal and the Antagonistic Tetrad

We posit that because Antagonistic Tetrad traits involve self and other focus, it is useful to understand how self-construal plays a role in shaping these traits. Narcissism features self-promoting, ego-reinforcement behaviour, which may be consistent with a more independent self-construal wherein individuals view themselves as unique and autonomous (Campbell et al., 2005). In line with this, Konrath et al. (2009) found that narcissism was positively related to an independent self-construal. Given its focus on self-enhancement and personal distinction, we hypothesize:

**H1a.**  *Independent self-construal will positively predict narcissism.*

**H2a.**  *Interdependent self-construal will negatively predict narcissism*.

Research on Machiavellianism has also highlighted its association with instrumental, self-focused social strategies. For example, Jonason et al. (2017) found that Machiavellianism was positively correlated with independence across samples from multiple countries. Although Robertson et al. (2016) reported no association between Machiavellianism and self-construal, their findings varied across cultural groups, suggesting the relationship may depend on broader sociocultural factors. Consistent with the emphasis on autonomy and strategic self-interest characteristic of Machiavellianism, we propose:

**H1b.**  *Independent self-construal will positively predict Machiavellianism.*

**H2b.**  *Interdependent self-construal will negatively predict Machiavellianism.*

Psychopathy is characterized by antisocial tendencies (Paulhus et al., 2021), which is inconsistent with a more interdependent self-construal that views the self as highly interconnected with others. In their review of the psychopathy literature, Lin and Xie (2023) report that dimensions of psychopathy such as risk-taking behaviour are associated with an independent self-construal, whereas social cohesion—reflective of an interdependent self-construal—is negatively associated with psychopathy. Based on this, we expect psychopathy to align more closely with independence and less with interdependence.

**H1c.**  *Independent self-construal will positively predict psychopathy*.

**H2c.**  *Interdependent self-construal will negatively predict psychopathy.*

Sadism is characterized by cruel behaviour intended to deliberately hurt others and assert dominance over others. Given that such behaviour is antithetical to a relationally focused, socially sensitive interdependent self-construal (Markus & Kitayama, 1991), we anticipate that sadism will follow a similar pattern to psychopathy.

**H1d.**  *Independent self-construal will positively predict sadism*.

**H2d.**  *Interdependent self-construal will negatively predict sadism*.

While Antagonistic Tetrad traits intuitively relate to how one views the self, little empirical research has examined these connections (see Jonason et al., 2017; Robertson et al., 2016), and existing findings are inconsistent. Notably, prior studies have focused on the Dark Triad and thus lack consideration of sadism as a fourth construct, further underscoring the need to examine how self-construal may predict the Antagonistic Tetrad.

In the present study, we recruited Canadian university students as participants. University samples are commonly used in research on self-construal and antagonistic personality traits, because they provide access to a large, relatively homogeneous population in terms of age and cognitive ability, while exhibiting meaningful variation in personality traits (Jonason et al., 2017; Robertson et al., 2016). Prior studies examining self-construal and antagonistic personality traits have frequently relied on student samples across diverse cultural contexts, including the United States, the Philippines, and European countries, demonstrating that such samples can meaningfully reflect individual differences in these constructs (Jonason et al., 2017; Konrath et al., 2009). By selecting a Canadian university sample, we were able to examine these associations in one of the world’s most culturally diverse contexts, extending prior work and allowing for a test of whether self-construal predicts antagonistic personality traits in a population characterized by multiple cultural backgrounds. 

## 2. Method

### 2.1. Participants and Procedure

Participants were 891 Canadian undergraduate business students. The sample was selected based on convenience through the University’s undergraduate research lab. Those with incomplete responses were removed, with all analyses conducted on the remaining 861 responses (57.7% men, 41.6% women, and 0.7% identified as nonbinary). Participants had a mean age of 18.21 years (*SD* = 1.13, range 16 to 37). Individuals completed the self-report measures described below on-line via Qualtrics and were given partial course credit for participating (not contingent on completion of the survey). The study received institutional ethical approval.

### 2.2. Measures

Participants provided demographic information, including age and identified gender (1 = men, 2 = women, 3 = nonbinary), which was initially re-coded to male (0/1), female (0/1), and nonbinary (0/1). Participants additionally completed the Singelis (1994) measure of independent and interdependent self-construal dimensions. This 24-item scale assesses two sub-scales, each with twelve items, responded to using 1 = strongly disagree, 2 = disagree, 3 = somewhat disagree, 4 = don’t agree or disagree, 5 = agree somewhat, 6 = agree, or 7 = strongly agree, response key. A sample independent scale item is, “I enjoy being unique and different from others in many respects.” A sample interdependent scale item is, “Even when I strongly disagree with group members, I avoid an argument.” For the present sample, Cronbach’s alpha for independence was 0.76 and 0.75 for interdependence.

Participants also completed the Paulhus et al. (2021) Short Dark Tetrad (SD4) scale as a measure of Antagonistic Tetrad traits, a 28-item scale that assesses four antagonistic personality traits, each with seven items, responded to using a 1 = strongly disagree, 2 = disagree, 3 = neutral, 4 = agree, 5 = strongly agree, response key. For the present data, Cronbach alpha for Machiavellianism was 0.70 (example item “It’s not wise to let people know your secrets”), 0.67 for narcissism (example item, “I have some exceptional qualities”), 0.74 for psychopathy (example item, “I tend to fight against authorities and the rules”), 0.79 for sadism (example item, “Some people deserve to suffer.”), and 0.87 for an Antagonistic Tetrad aggregate. While the narcissism subscale alpha estimate in the present study is slightly lower than conventional thresholds, it is consistent with prior research using this brief scale (Paulhus et al., 2021) and acceptable given the small number of items and the mean inter-item correlations (0.24–0.35) indicative of adequate internal consistency (Briggs & Cheek, 1986).

## 3. Results

### 3.1. Correlations

Table 1 provides the zero-order correlations between the study variables. In terms of demographics, men scored significantly higher than women on each of the Antagonistic Tetrad dimensions. The antagonistic trait sub-scales had moderate inter-correlations, ranging from 0.33 to 0.60. Men scored lower in interdependence and higher in independence than women. With respect to self-construal, the independent and interdependent self-construal scales are moderately positively correlated, consistent with recent evidence that these dimensions can co-occur within individuals.

### 3.2. Joint Factor Analyses of Antagonistic Tetrad Scores and Self-Construal

To further investigate the relationship between the personality dimensions of the Antagonistic Tetrad and self-construal, joint factor analyses were conducted for the complete sample and then for men and women separately (given that gender showed significant associations with the study variables, as reported in Table 1). Table 2 presents the pattern matrix factor loadings for the self-construal and Antagonistic Tetrad scales following principal axis factoring with oblimin rotation. For each analysis, two factors were extracted based on the scree plot and eigenvalue-greater-than-unity criteria. Of interest, for both the full sample and the men-only sample, the self-construal scales loaded strongly on one factor, whereas the Antagonistic Tetrad scales loaded on a second factor, with minimal cross-loadings between constructs. This pattern suggests a relatively clear structural distinction between self-construal and antagonistic personality traits in these groups. In contrast, for women, narcissism loaded on both factors and demonstrated a stronger loading on the self-construal factor than on the Antagonistic Tetrad factor. This pattern suggests that, for women, narcissism may be more closely tied to self-related and self-evaluative processes reflected in self-construal, rather than functioning solely as an antagonistic trait. In other words, these results indicate that the latent structure linking self-construal and antagonistic traits differs by gender, with narcissism occupying a more hybrid or self-oriented role among women compared to men.

### 3.3. Predicting Antagonistic Tetrad Scores

We conducted preliminary analyses to consider the appropriate means of including gender in the analysis by entering each of independent and interdependent self-construal, age, and the nonbinary variable as predictors of the Antagonistic Tetrad. As the non-binary variable was not a significant predictor in any of the models (*p* = 0.11) we subsequently restricted remaining analyses to gender coded as men (1) and women (2). Age and gender were included as control variables in subsequent analyses given evidence that both can influence antagonistic traits (Jonason et al., 2017; Muris et al., 2017; Paulhus & Williams, 2002), allowing us to examine the unique predictive effects of self-construal.

**Figure 1:**
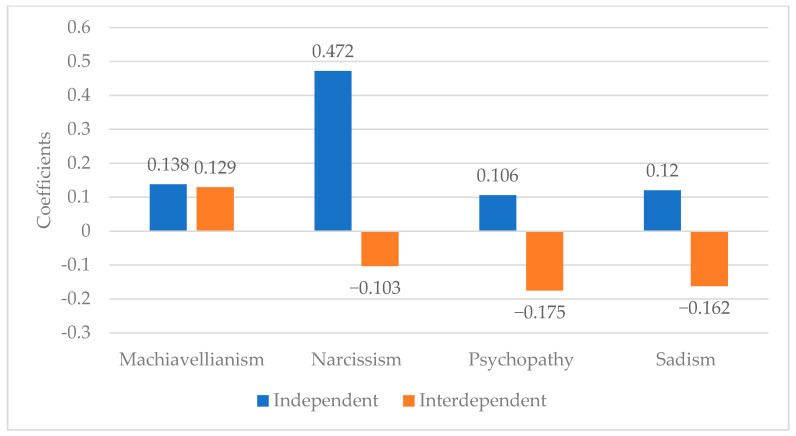
Standardized Regression Coefficients for Self-Construal Predicting Antagonistic Traits.

Direct-entry linear regression analyses were conducted to predict each of the antagonistic dimension scores as well as the overall Antagonistic Tetrad aggregate scale. To address potential concerns about multicollinearity in our regression models, we examined the variance inflation factor (VIF) for each predictor. All VIFs were below the commonly used threshold of 5 (range = 1.008–1.269), indicating that collinearity was not likely to have inflated standard errors or obscured effects. Table 3 lists the regression results which reveal a nuanced relationship between self-construal dimensions and the antagonistic traits and Figure 1 provides a visual of the regression coefficients.

In line with hypotheses, narcissism, psychopathy, and sadism were each positively predicted by independent self-construal and negatively predicted by interdependent self-construal. These patterns suggest that these antagonistic traits are grounded in a self-focused, autonomous orientation and are inhibited by a relational, socially attuned worldview. Machiavellianism, in contrast, was predicted positively by both independent and interdependent self-construals. This unique finding suggests that Machiavellianism may involve a dual motivational foundation—driven by both an autonomous strategic calculation and by a relational awareness that facilitates social manipulation. This finding is particularly notable as it sets Machiavellianism apart from the other traits.

The variance explained by self-construal was notably greater for narcissism and sadism than for psychopathy and Machiavellianism. This suggests that self-construal exerts a stronger influence on antagonistic traits rooted in ego-enhancement and affective response (narcissism, sadism) when compared to those marked by strategic behaviour (Machiavellianism) or impulsiveness (psychopathy). Gender consistently predicted all four traits, with females reporting lower scores across the board, supporting literature that associates male gender with higher antagonistic characteristics (Gómez-Leal et al., 2024). These results collectively indicate that while the antagonistic traits share some common self-construal antecedents, they also diverge in theoretically meaningful ways.

## 4. Discussion

This research contributes to the understanding of the Antagonistic Tetrad and the predictive role of self-construal in explaining individual’s antagonistic personality traits. Most notably, the negative relationship between interdependence and sadism is an important finding given that previous research examining correlations between self-construal and the Dark Triad has not considered the role of sadism. Demonstrating that self-construal dimensions can predict these traits suggests that cultural and self-perceptual frameworks play a role in the development or expression of malevolent personality features. Specifically, an independent self-construal, which values autonomy and self-interest, may be more conducive to the emergence of antagonistic traits, whereas an interdependent self-construal may serve as a protective factor against them.

This study extends the literature on malevolent personality traits by examining the role of self-construal in predicting antagonistic traits—narcissism, Machiavellianism, psychopathy, and sadism. While previous research has largely focused on the Dark Triad (e.g., Jonason et al., 2017; Robertson et al., 2016), our inclusion of sadism reflects that it constitutes a distinct component of antagonistic personality (Paulhus, 2014; Buckels et al., 2013; Kay et al., 2025). By incorporating sadism and demonstrating its negative association with interdependence, this study expands the scope of self-construal research beyond what has been empirically established.

We found that independent self-construal positively predicted all four antagonistic traits examined. This is consistent with theories suggesting that an autonomous and self-focused orientation fosters behaviours associated with self-enhancement, social dominance, and diminished regard for others (Cross et al., 2011). Individuals who construe themselves as unique and independent are more likely to express traits such as narcissism and sadism, which involve egocentrism and even pleasure from inflicting harm (Buckels et al., 2013). Conversely, interdependent self-construal negatively predicted narcissism, psychopathy, and sadism, but not Machiavellianism. This aligns with evidence that interdependent individuals are more likely to engage in empathic, prosocial behaviour and to inhibit traits that disrupt group harmony (Markus & Kitayama, 1991; Cross et al., 2011). These results support the view that interdependence can act as a psychological buffer against more antagonistic or harmful personality expressions.

Notably, Machiavellianism was positively predicted by both independent and interdependent self-construal, suggesting a more complex dual-process foundation. This might reflect the strategic flexibility of Machiavellian individuals, who can manipulate both individualistic and relational contexts to their advantage (Jones & Paulhus, 2009). Unlike the impulsivity seen in psychopathy or the ego-centricity of narcissism, Machiavellianism may involve a more instrumental social intelligence that can be compatible with both self-orientations. The finding that self-construal explained greater variance in narcissism and sadism than in psychopathy and Machiavellianism suggests that self-perception has stronger links to traits involving ego-gratification and emotional engagement than to those characterized by strategic or behavioural tendencies (Jones & Figueredo, 2013).

Beyond the regression findings, the gender-specific factor analyses provide additional insight into how self-construal and antagonistic traits are structurally related. The structural gender distinctions may help explain why narcissism often shows weaker or more inconsistent relations with antisociality than other antagonistic traits and highlights the importance of considering gender when interpreting the psychological meaning of narcissism. Together, these findings suggest that the links between self-construal and antagonistic traits are not only trait-specific but also gender-contingent, underscoring the value of examining latent structure.

Although independence and interdependence are often conceptualized as opposing constructs (Markus & Kitayama, 1991), more recent empirical work has shown that they can be positively related within individuals (e.g., Vignoles et al., 2016; Harb & Smith, 2008). That is, some individuals may strongly endorse both self-reliance and relational connectedness, reflecting a multidimensional, rather than bipolar, structure of self-construal. This might be particularly likely in a multicultural country like that sampled for this study, Canada. Consistent with this, we observed a significant positive correlation between independence and interdependence.

These findings deepen our understanding of the cultural and cognitive underpinnings of Antagonistic Tetrad traits. By identifying differential self-construal patterns across traits, this study contributes to a more nuanced, trait-specific, and culturally informed framework for understanding personality pathology. In particularly, it highlights the divergent self-construal foundations of antagonistic traits, clarifies the dual motivation of Machiavellianism; and extends the work of Robertson et al. (2016) and Jonason et al. (2017) by incorporating sadism and demonstrating that interdependent self-construal negatively predicts antisociality, a relationship not clearly established in their work. Collectively these contributions support a culturally informed understanding of personality, where independent and interdependent worldviews shape the expression of antagonistic traits.

### 4.1. Limitations and Future Directions

This research has several limitations worthy of note. As is typical of an undergraduate student sample, the participants may not be representative of the general population. Further, given that self-construal is influenced by culture (Markus & Kitayama, 1991), it is important for interpretation of the results to note that the sample is Canadian, and therefore replication of these findings with an additional independent sample would further test the robustness and generalizability of the present findings. Another limitation concerns our use of the Short Dark Tetrad (SD4) scale, which, while efficient for large-scale research, provides brief assessments of antagonistic personality traits that do not fully reflect the multidimensionality of each trait (Jones & Paulhus, 2014). Relatedly, prior work has debated regarding overlap between the SD4 measures of sadism and psychopathy (e.g., Blötner & Mokros, 2023; Kay et al., 2025), which constrains the ability to draw strong conclusions about sadism as a distinct construct.

Our reliance on a single self-report source also introduces the possibility of common method bias (Podsakoff et al., 2003). The present study is also limited in that the order of the scales was fixed which may have influenced responses. Future research could address these issues by employing longer, multidimensional assessments of each antagonistic trait, randomizing the order of scales or items, and by incorporating multi-method designs—such as informant ratings, behavioral tasks, or longitudinal approaches—to provide a more comprehensive and methodologically robust test of the relationships observed in this study. In line with this, our conceptualization of self-construal as a predictor implies but does not support causality, i.e., the correlational nature of this research means that the traits might just as plausibly predict self-construal as the reverse. We highlight that the theoretical framing of self-construal as an antecedent rests on decades of research showing that self-construal shapes cognitive, motivational, and identity processes (Markus & Kitayama, 1991; Singelis, 1994), functioning upstream in the psychological architecture. For instance, independent self-construal promotes approach motivation and cognitive flexibility, thereby influencing creative behavior (Shao et al., 2019). Self-construal also contributes to the structure and stability of identity, supplying a foundation upon which traits and self-concept emerge (Vignoles et al., 2016). At the same time, bidirectional influences are certainly plausible— antagonistic traits may shape how people construe themselves, for example through altered identity coherence or social regulation styles.

In sum, because the personality traits assessed in this research are considered relatively stable individual differences, and such traits are often resistant to short-term change on self-report measures (Roberts et al., 2017) it may be that a laboratory manipulation of self-construal design may not be effective in shifting personality self-reports. Stronger designs—such as longitudinal studies tracking changes in self-construal alongside personality, or multi-session interventions combining self-construal priming with sustained social or behavioural experiences—could perhaps more meaningfully test causal pathways.

### 4.2. Conclusions

This study is the first to systematically examine how independent and interdependent self-construals predict each trait within the Antagonistic Tetrad, offering novel insights into the cultural and psychological foundations of malevolent personality. These findings advance our understanding of how self-perception and cultural models of the self shape the manifestation of personality traits. In particular, the protective role of interdependence against psychopathy and sadism introduces a valuable dimension to the literature, with practical implications for intervention, leadership selection, and cross-cultural assessment. By identifying the differential predictive utility of self-construal across the Antagonistic Tetrad, this study lays the groundwork for future research to investigate how these relationships unfold in various sociocultural contexts and behavioural domains, including aggression, manipulation, consumption, and ethical decision-making.

## Figures and Tables

**Table 1:** Correlations.

	1.	2.	3.	4.	5.	6.	7.	8.	9.	10.
1. Nonbinary	--									
2. Female	−0.07 *	--								
3. Male	−0.10 *	−0.99 **	--							
4. Age	−0.01	−0.08 *	0.08 *	--						
5. Interdependence	−0.01 **	0.10 **	−0.09 *	−0.06	--					
6. Independence	−0.05	−0.06	0.08 *	−0.05	0.43 **	--				
7. Machiavellianism	−0.05	−0.12 **	0.11 **	0.01	0.17 **	0.20 **	--			
8. Narcissism	−0.04	−0.11 **	0.10 **	−0.07	0.08 *	0.43 **	0.41 **	--		
9. Psychopathy	−0.05	−0.27 **	0.26 **	0.05	−0.17 **	0.03	0.33 **	0.37 **	--	
10. Sadism	−0.05	−0.48 **	0.47 **	−0.01	−0.15 **	0.09 *	0.40 **	0.33 **	0.60 **	--
11. Tetrad	−0.06	−0.35 **	0.34 **	−0.01	−0.04	0.22 **	0.70 **	0.66 **	0.79 **	0.83 **
Mean				18.21	57.83	57.81	24.38	19.18	16.25	19.12
SD				1.13	8.72	9.27	4.18	3.60	4.55	5.60

*Note*: *N* = 861; ** *p* < 0.001; * *p* < 0.05, two-tailed.

**Table 2:** Joint Factor Analyses.

	All Factor I	All Factor II	Men Factor I	Men Factor II	Women Factor I	Women Factor II
Interdependence	−0.14	0.64	−0.13	−0.73	−0.19	0.40
Independence	0.13	0.74	0.07	−0.82	0.08	0.59
Machiavellianism	0.51	0.24	0.42	−0.35	0.55	0.18
Narcissism	0.53	0.34	0.58	−0.31	0.46	0.52
Psychopathy	0.77	−0.18	0.70	0.17	0.76	−0.08
Sadism	0.78	−0.15	0.72	0.06	0.82	−0.21
KMO	0.653		0.652		0.618	
Percentage of Variance	65.93		66.78		62.55	
Correlation between Factors I and II	0.143		−0.20		0.06	

Values in table represent Pattern Matrix factor loadings following Principal Axis Factoring with Oblimin Rotation; KMO = Kaiser-Meyer-Olkin Measure of Sampling Adequacy.

**Table 3:** Regression results.

	Machiavellianism		Narcissism		Psychopathy		Sadism	
Predictor	Beta	*t*	Beta	*t*	Beta	*t*	Beta	*t*
Independence	0.138	3.71 **	0.472	13.74 **	0.106	2.89 *	0.120	3.60 **
Interdependence	0.129	3.46 **	−0.103	−2.98 *	−0.175	−4.77 **	−0.162	−4.86 **
Gender	−0.122	−3.57 **	−0.064	−2.02 *	−0.244	−7.28 **	−0.460	−15.13 **
Age	0.010	0.287	−0.054	−1.74	0.017	0.508	−0.051	−1.71
	*F*(4,830) = 14.43 **		*F*(4,830) = 53.98 **		*F*(4,830) = 22.76 **		*F*(4,830) = 71.70 **	
	Adjusted *R*^2^ = 0.06		Adjusted *R*^2^ = 0.20		Adjusted *R*^2^ = 0.10		Adjusted *R*^2^ = 0.25	

*Notes*: Gender is coded 1 = men, 2 = women; ** *p* < 0.001; * *p* < 0.05.

## Data Availability

Data is available at https://osf.io/xspb4/files/ze3wt (Uploaded 14 April 2023).
